# Supplementary material for: Coenzyme A biosynthesis in Bacillus subtilis: discovery of a novel precursor metabolite for salvage and its uptake system
Source: mBio. 2024 Aug 28;15(10):e01772-24. doi: 10.1128/mbio.01772-24 (PMC11487621; doi:10.1128/mbio.01772-24)
Supplement: Supplemental Figures — Figures S1 to S6. [file mbio.01772-24-s0001.pdf]

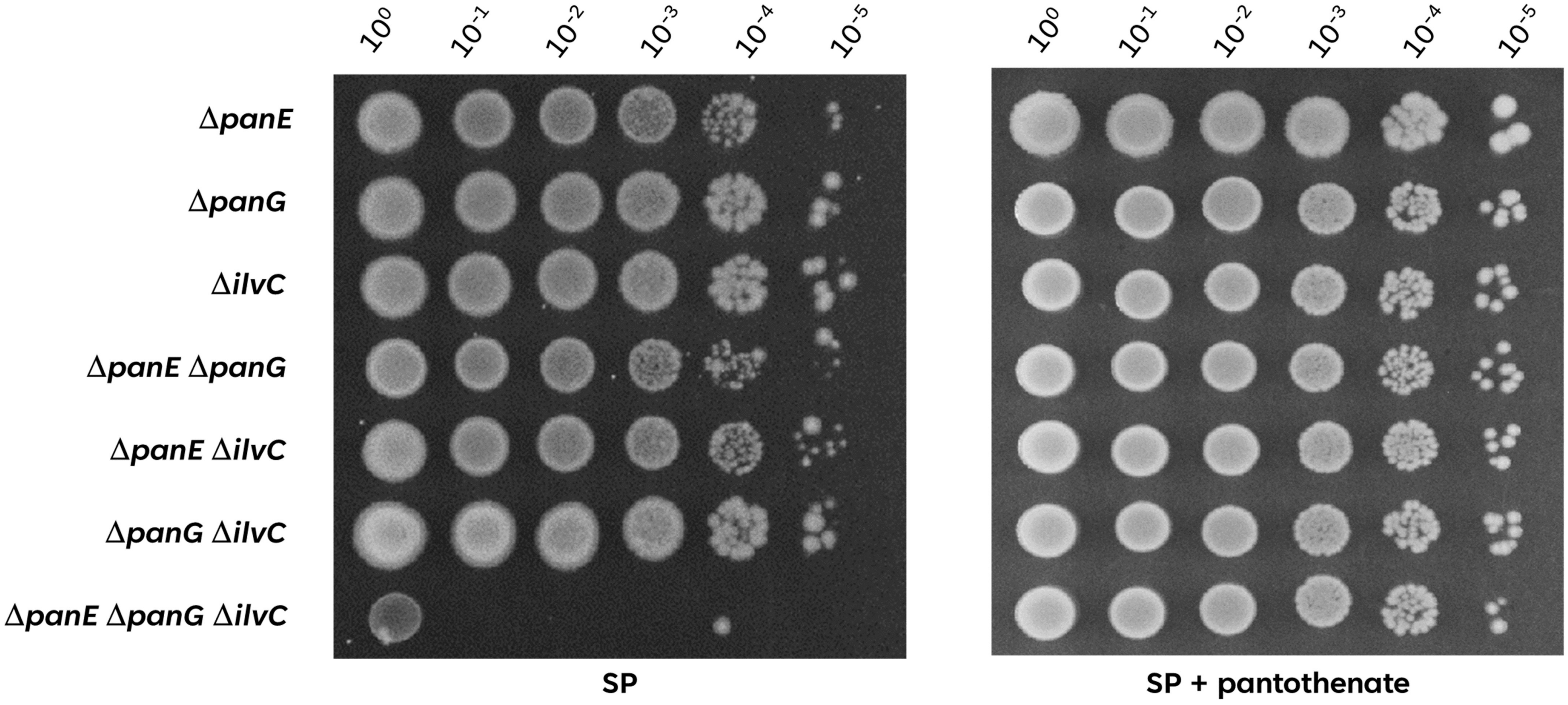

Figure S1: The three enzymes PanE, PanG and IlvC are involved in pantoate biosynthesis. The growth of the *B. subtilis* deletion mutants Δ*panE* (GP3384), Δ*panG* (GP3383), Δ*ilvC* (GP4404), Δ*panE* Δ*panG* (GP3343), Δ*panE* Δ*ilvC* (GP3397), Δ*panG* Δ*ilvC* (GP3346) and Δ*panE* Δ*panG* Δ*ilvC* (GP4403) was compared. The cells were grown in sporulation medium (SP) supplemented with 1 mM pantothenate to an OD_{600} of 1.0, and serial dilutions (10-fold) were prepared. These samples were plated on SP with or without added 1 mM pantothenate and incubated at 37° C for 48 h.

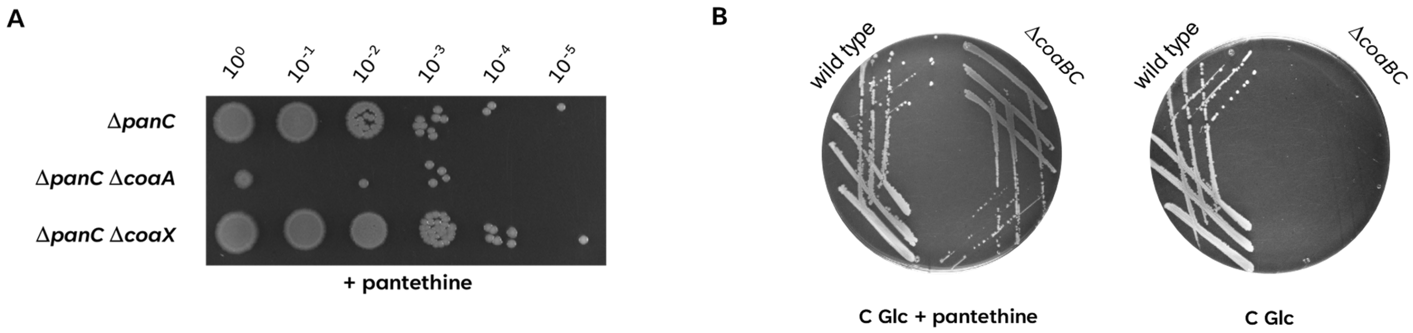

Figure S2: *B. subtilis* harbors a CoaA- and pantetheine-dependent *coaBC* bypass of the biosynthetic pathway. A, The growth of the Δ*panC* mutant (GP4379) was compared with the isogenic Δ*panC* Δ*coaA* (GP4652) and Δ*panC* Δ*coaX* (GP4653) mutants. The cells were grown in sporulation medium (SP) supplemented with 1 mM pantothenate to an OD_{600} of 1.0, and serial dilutions (10-fold) were prepared. These samples were plated on C Glc minimal medium supplemented with 250 µM pantethine and incubated at 37° C for 48 h. B, The deletion mutant Δ*coaBC* GP4670 was streaked out on C Glc minimal medium plates with or without added 250 µM pantethine. The plates were incubated at 37° C for 24h.

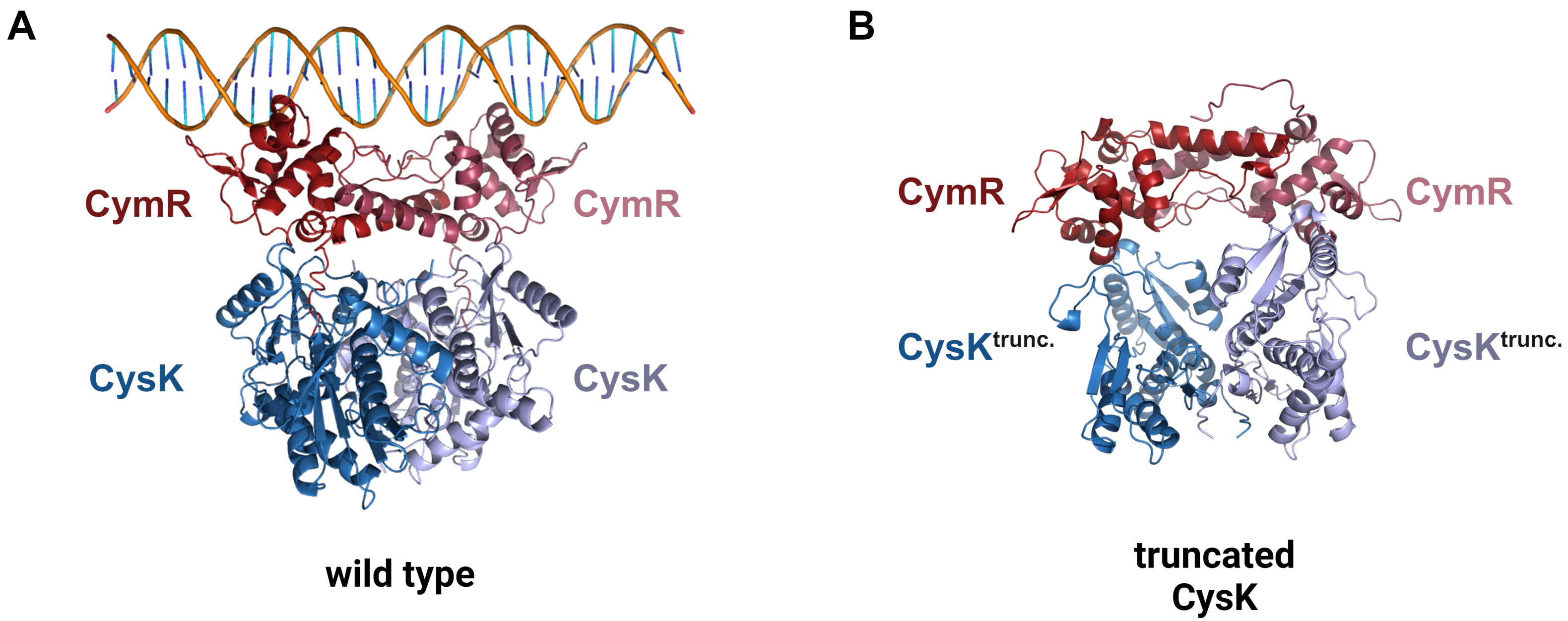

Figure S3: Effect of the CysK truncation on the interaction with CymR. ColabFold/ AlphaFold predicted model of Cym dimer (red, purple) in complex with the full length (A) and truncated (B) CysK dimer (light blue, dark blue). For the wild type model (A), the 5’ UTR of the *cysK* gene was docked with HDOCK.

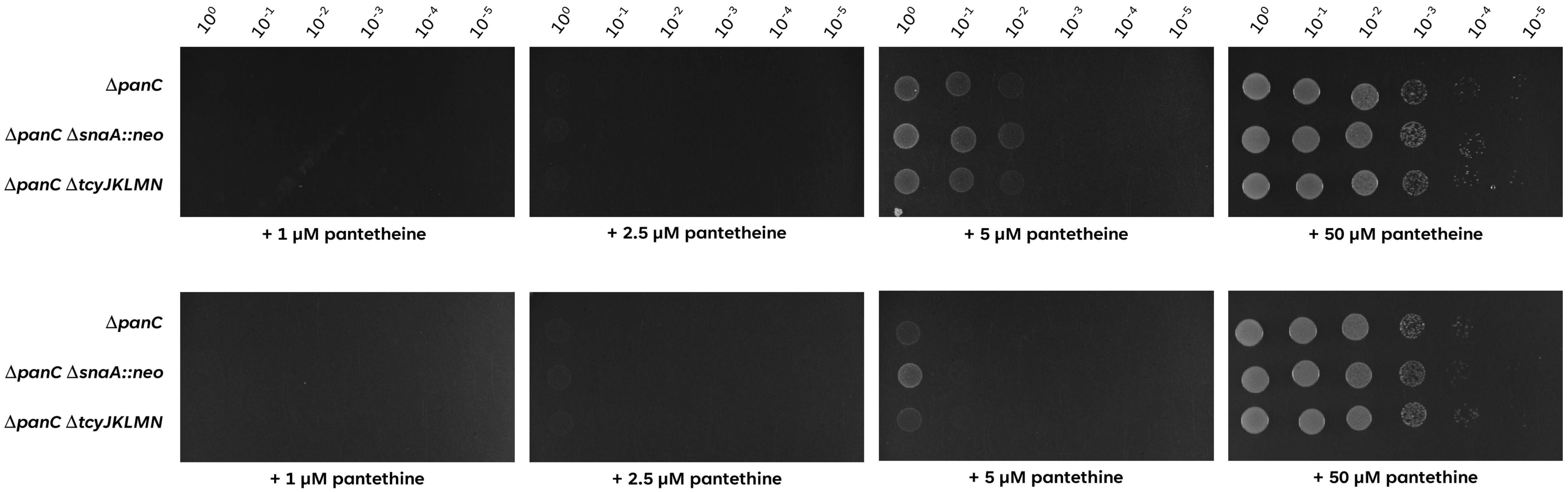

Figure S4: TcyJKLMN is not active in the transport of pantetheine or pantethine. The growth of the Δ*panC* mutant (GP4361) was compared with the isogenic Δ*panC* Δ*snaA::neo* (GP4364) and Δ*panC* Δ*tcyJKLMN* (GP4660), overexpressing or lacking the ABC transporter TcyJKLMN respectively. The cells were grown in C Glc minimal medium supplemented with 1 mM pantothenate to an OD_{600} of 1.0, and serial dilutions (10-fold) were prepared. These samples were plated on C Glc minimal medium plates supplemented with 1 µM, 2.5 µM, 5 µM or 50 µM of either pantetheine or pantethine. The plates were incubated at 37° C for 24h.

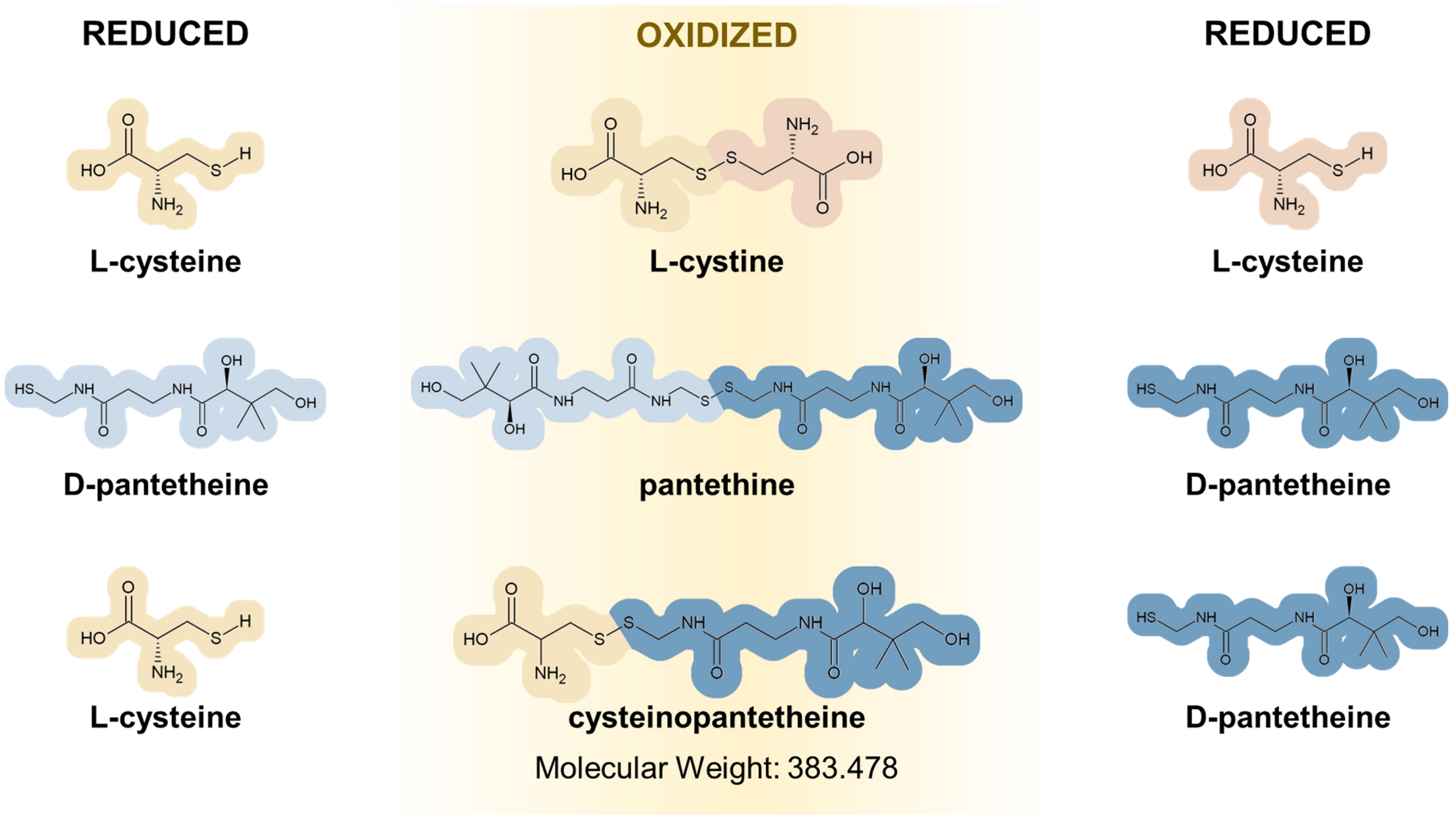

Figure S5: Molecular Structures of the reduced monomers L-cysteine and D-pantetheine and their oxidized dimeric forms L-cystine, pantethine and cysteinopantetheine. The moieties are colorized to enhance the visibility of the dimeric form, for cysteine in dark/light yellow and pantetheine in dark/light blue. Two reduced molecules (left and right panels) can form one oxidized molecule via formation of sulfur bridges (middle panel).

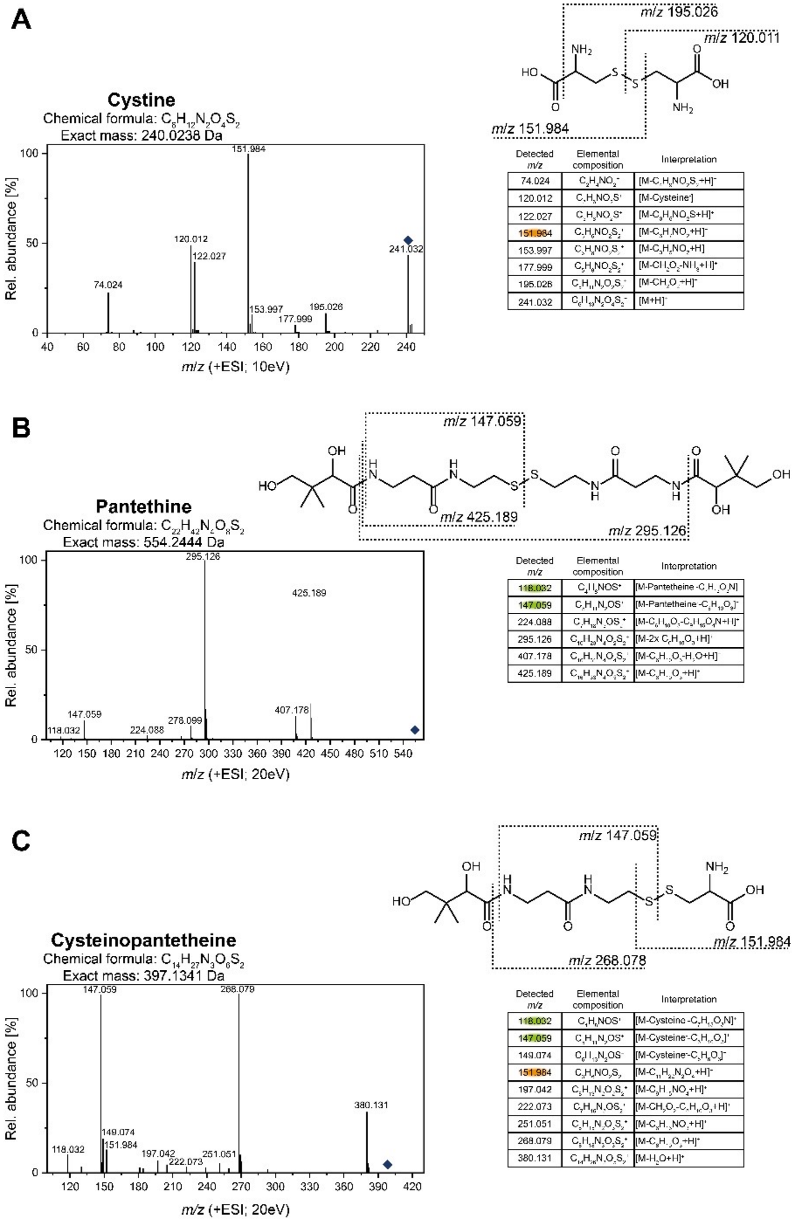

Figure S6: The identity of cysteinopantetheine was confirmed by comparative MS/MS analyses with cystine and pantethine. Authentic standards for (A) cystine and (B) pantethine as well as (C) a solution containing cysteinopantetheine (generated by incubation of cysteine with pantetheine, see Materials and Methods) were analyzed by ultra-high performance liquid chromatography coupled to high resolution mass spectrometry and fragmented by collision induced dissociation at 10 eV (A) or 20 eV (B,C) in positive ionization mode. The respective MS/MS spectra were interpreted as shown in the tables and as characteristic fragments in the structure. A cysteine moiety is considered as $C_3H_6NO_2S$ and a pantetheine moiety is considered as $C_{11}H_{21}N_2O_8S$. The respective precursor ions are marked by a rhomb. Characteristic fragments used for structure confirmation of cysteinopantetheine are marked in orange and green, respectively.
