# Supplementary material for: Coenzyme A biosynthesis in Bacillus subtilis: discovery of a novel precursor metabolite for salvage and its uptake system
Source: mBio. 2024 Aug 28;15(10):e01772-24. doi: 10.1128/mbio.01772-24 (PMC11487621; doi:10.1128/mbio.01772-24)
Supplement: Legend — for Movie S1. [file mbio.01772-24-s0002.pdf]

Supplemental Movie - Movie SM1

The interaction between CysK and CymR triggers DNA binding by CymR. In the absence of cysteine, apo-CymR forms an inactive hexamer. In the presence of cysteine, the cysteine synthase CysK binds to CymR, resulting in the dissociation of the hexamer to a CymR2-CysK2 heterotetramer. This complex binds its DNA targets to repress the genes and operons of the CymR regulon. The model is based on the crystal structure of apo-CymR and AlphaFold multimer predictions of CymR-CysK complex formation as well as HDOCK modeling of the CymR-DNA interaction.
