# Supplementary material for: Coenzyme A biosynthesis in Bacillus subtilis: discovery of a novel precursor metabolite for salvage and its uptake system
Source: mBio. 2024 Aug 28;15(10):e01772-24. doi: 10.1128/mbio.01772-24 (PMC11487621; doi:10.1128/mbio.01772-24)
Supplement: Table S1 — Bacterial strains used in this study. [file mbio.01772-24-s0003.pdf]

Table S1. ***B. subtilis*** **strains used in this study**

**Strain**	**Genotype**	**Reference**
168	*trpC2*	Laboratory collection
BKK00700	*trpC2 ΔcoaX::neo*	13
BKK00730	*trpC2 ΔcysK::neo*	13
BKK01470	*trpC2 ΔecfT::neo*	13
BKK04520	*trpC2 ΔydbM::neo* [1]	13
BKK07190	*trpC2 ΔyezD::neo* [1]	13
BKK07200	*trpC2 ΔyetJ::neo* [1]	13
BKK09130	*trpC2 ΔtcyP::neo* [1]	13
BKK10010	*trpC2 ΔtrpP::neo*	13
BKK10370	*trpC2 ΔpanU:neo*	13
BKK14440	*trpC2 ΔpanG::neo*	13
BKK15110	*trpC2 ΔpanE::neo*	13
BKK15580	*trpC2 ΔcysP::neo* [1]	13
BKK15610	*trpC2 ΔylnD::neo* [1]	13
BKK15620	*trpC2 ΔsirB::neo* [1]	13
BKK15630	*trpC2 ΔylnF::neo* [1]	13

BKK21980	*trpC2 ΔypdP::neo*	13
BKK22410	*trpC2 ΔpanD::neo*	13
BKK22420	*trpC2 ΔpanC::neo*	13
BKK22430	*trpC2 ΔpanB::neo*	13
BKK23050	*trpC2 ΔribU::neo*	13
BKK23760	*trpC2 ΔcoaA::neo*	13
BKK27240	*trpC2 ΔyrhC::neo* [1]	13
BKK27260	*trpC2 ΔmccA::neo* [1]	13
BKK27270	*trpC2 ΔmtnN::neo* [1]	13
BKK27280	*trpC2 ΔyrrT::neo* [1]	13
BKK27520	*trpC2 ΔcymR::neo*	13
BKK28290	*trpC2 ΔilvC::neo*	13
BKK29280	*trpC2 ΔytnM::neo* [1]	13
BKK29290	*trpC2 ΔsndA::neo* [1]	13
BKK29300	*trpC2 ΔribR::neo* [1]	13
BKK29330	*trpC2 ΔcmoO::neo* [1]	13
BKK29340	*trpC2 ΔtcyN::neo* [1]	13
BKK29350	*trpC2 ΔtcyM::neo* [1]	13
BKK29360	*trpC2 ΔtcyL::neo* [1]	13

BKK29390	*trpC2 ΔsnaA::neo*	13
BKK29400	*trpC2 ΔascR::neo* [1]	13
BKK30990	*trpC2 ΔthiT::neo*	13
BKK32030	*trpC2 ΔyuiG::neo*	13
BKK39460	*trpC2 ΔyxeQ::neo* [1]	13
BKK39470	*trpC2 ΔsndB::neo* [1]	13
BKK39490	*trpC2 ΔyxeN::neo* [1]	13
BKK39510	*trpC2 ΔsnaB::neo* [1]	13
BKK39520	*trpC2 ΔyxeK::neo* [1]	13
GP1171	*trpC2 xkdE::(N-yfp erm^R)*	pGP886 → 168
GP3342	*trpC2 ΔilvC::lox72*	pDR244 → GP4404 (heat cured)
GP3343	*trpC2 ΔpanG::lox72 ΔpanE::lox72*	pDR244 → GP3348 (heat cured)
GP3346	*trpC2 ΔpanG::lox72 ΔilvC::neo*	BKK28290 → GP3347
GP3347	*trpC2 ΔpanG::lox72*	pDR244 → GP3383 (heat cured)
GP3348	*trpC2 ΔpanG::lox72 ΔpanE::neo*	BKK15110 → GP3347
GP3380	*trpC2 ΔcoaA::neo*	BKK23760 → 168
GP3381	*trpC2 ΔcoaX::neo*	BKK00700 → 168

GP3382	*trpC2 ΔpanD::neo*	BKK22410 → 168
GP3383	*trpC2 ΔpanG::neo*	BKK14440 → 168
GP3384	*trpC2 ΔpanE::neo*	BKK15110 → 168
GP3386	*trpC2 ΔcysK::neo*	BKK00730 → 168
GP3397	*trpC2 ΔilvC::lox72 ΔpanE::neo*	GP3384 → GP3342
GP4085	*trpC2 ΔcymR::neo*	BKK27520 → 168
GP4124	*trpC2 ΔcysK::lox72 ΔpanB::neo*	GP4401 → GP4462
GP4361	*trpC2 ΔpanC::cat*	This study
GP4362	*trpC2 ΔpanC::cat ΔcymR::lox72*	GP4361 → GP4463
GP4364	*trpC2 ΔpanC::cat ΔsnaA::neo*	GP4361 → GP4460
GP4375	*trpC2 ΔpanC::cat ΔcymR::lox72 ΔtcyN::neo*	BKK29340 → GP4362
GP4378	*trpC2 ΔpanC::cat ΔtcyN::neo*	BKK29340 → GP4361
GP4379	*trpC2 ΔpanC::cat xkdE::Pxyl-(empty)-ermC*	GP1171 → GP4361
GP4380	*trpC2 ΔpanC::cat xkdE::Pxyl-tcyJKLMN-ermC*	pGP4018 (ScaI) → GP4361
GP4383	*trpC2 ΔpanC::cat ΔsnaA::lox72*	pDR244 → GP4364 (heat cured)
GP4401	*trpC2 ΔpanB::neo*	BKK22430 → 168
GP4402	*trpC2 ΔpanC::neo*	BKK22420 → 168
GP4403	*trpC2 ΔpanG::lox72 ΔpanE::lox72 ΔilvC::neo*	BKK28290 → GP3343

GP4404	*trpC2 ΔilvC::neo*	BKK28290 → 168
GP4444	*trpC2 ΔpanC::cat xkdE::Pxyl-tcyJKLMN-ermC ΔcoaA::neo*	GP3380 → GP4380
GP4448	*trpC2 ΔpanB::neo* $cysK^{G223*}$ $alaS^{P731L}$	GP4401 suppressor on SP (pantothenate depletion)
GP4449	*trpC2 ΔpanB::neo* $cysK^{G223*}$	GP4401 suppressor on SP (pantothenate depletion)
GP4450	*trpC2 ΔpanC::neo* $guaB^{D64IAMA}$ *[ΔydcL-yddM]* $yncM^{K13E}$ $cymR^{L49T}$	GP4402 suppressor on SP (pantothenate depletion)
GP4460	*trpC2 ΔsnaA::neo*	BKK29390 → 168
GP4462	*trpC2 ΔcysK::lox72*	pDR244 → GP3386 (heat cured)
GP4463	*trpC2 ΔcymR::lox72*	pDR244 → GP4085 (heat cured)
GP4489	*trpC2 ΔcoaX::neo xkdE::Pxyl-tcyJKLMN-ermC ΔpanC::cat*	GP3381 → GP4380
GP4652	*trpC2 ΔpanC::cat xkdE::Pxyl-(empty)-ermC ΔcoaA::neo*	GP3380 → GP4379
GP4653	*trpC2 ΔpanC::cat xkdE::Pxyl-(empty)-ermC ΔcoaX::neo*	GP3381 → GP4379
GP4659	*trpC2 ΔtcyJKLMN::tet*	This study

GP4660	*trpC2 ΔpanC::cat ΔtcyJKLMN::tet*	GP4659 → GP4361
GP4670	*trpC2 ΔcoaBC::spc*	This study
GP4694	*trpC2 ΔpanC::cat ΔribU::neo*	BKK23050 → GP4361
GP4695	*trpC2 ΔpanC::cat ΔthiT::neo*	BKK30990 → GP4361
GP4696	*trpC2 ΔpanC::cat ΔtrpP::neo*	BKK10010 → GP4361
GP4697	*trpC2 ΔpanC::cat ΔpanU::neo*	BKK10370 → GP4361
GP4698	*trpC2 ΔpanC::cat ΔypdP::neo*	BKK21980 → GP4361
GP4699	*trpC2 ΔpanC::cat ΔyuiG::neo*	BKK32030 → GP4361
GP4700	*trpC2 ΔpanC::cat ΔecfT::neo*	BKK01470 → GP4361
GP4703	*trpC2 ΔecfT::neo*	BKK01470 → 168
GP4704	*trpC2 ΔpanU::neo*	BKK10370 → 168

[1] These mutants were used in the initial screening to elucidate the suppression effect.
